# From perceptual to language-mediated categorization

**DOI:** 10.1098/rstb.2012.0391

**Published:** 2014-01-19

**Authors:** Gert Westermann, Denis Mareschal

**Affiliations:** 1 Department of Psychology, Lancaster University, Lancaster LA1 4YW, UK; 2 Centre for Brain and Cognitive Development, Birkbeck College, University of London, London WC1E 7HX, UK

**Keywords:** infant categorization, cognitive development, computational modelling, neural networks, learning

## Abstract

From at least two months onwards, infants can form perceptual categories. During the first year of life, object knowledge develops from the ability to represent individual object features to representing correlations between attributes and to integrate information from different sources. At the end of the first year, these representations are shaped by labels, opening the way to conceptual knowledge. Here, we review the development of object knowledge and object categorization over the first year of life. We then present an artificial neural network model that models the transition from early perceptual categorization to categories mediated by labels. The model informs a current debate on the role of labels in object categorization by suggesting that although labels do not act as object features they nevertheless affect perceived similarity of perceptually distinct objects sharing the same label. The model presents the first step of an integrated account from early perceptual categorization to language-based concept learning.

## 1. Introduction

During their first 2 years of life, infants move from the vast array of seemingly disconnected sensory experiences towards a sophisticated knowledge of objects, people and events, including the ability to group perceptually different objects into common categories, and to understand and produce the names for many of them [1,2]. Two closely related areas of research have addressed infants’ developing knowledge about individual objects, and the ability to form object categories, respectively. Research on object knowledge has investigated the progressive increase in the complexity of infants’ representation of objects, for example the developing ability to encode correlations between object features, and the ability to link information from different sensory domains. Work on object categorization has asked how infants group perceptually distinct objects together and how the basis for these categories changes across development.

Research on early word learning has likewise proceeded along separate strands. One strand, usually concerned with infants and toddlers from 14 months of age, has focused on when and how words are learned, how they are linked to objects, how they are used and extended to novel objects and how knowledge of a label affects children's inferences about an object's hidden properties. A second strand, usually focusing on younger infants around 10–12 months of age, addresses the question of how labels affect infants’ categorization of objects that share the same label or that are labelled differently.

A coherent account of early semantic development should encompass all of these research fields. Furthermore, it is important to acknowledge that, while many studies test infants’ abilities to acquire object knowledge in laboratory-based experimental settings, the knowledge shown by infants in such settings arises from an interaction between what was learned in the laboratory and prior knowledge acquired outside the laboratory. In this paper, we aim to present a first step towards an integrated account of semantic development in the form of a computer model that accounts for phenomena in early prelinguistic categorization as well as for the role of word learning on category structures. Computational models are useful tools for developing and testing hypotheses of the mechanisms underlying development and for linking individual observations of developmental abilities into a coherent trajectory of developmental change [3–5].

In the rest of this paper, we first review the development of object knowledge and categorization, characterizing both as shaped by a progressive development in the ability to integrate aspects of information leading to the enrichment of category representations. In the second part of the paper, we then present a computational model that has previously been used to account for apparently contradictory results in early object categorization, and we enhance it to account for the transition from prelinguistic to language-mediated object categorization.

## 2. The development of object categorization

The ability to categorize lies at the heart of semantic development. Knowledge of categories not only carves up the world in meaningful ways, but it also enables us to infer and predict properties of newly encountered objects. For example, by possessing the category ‘dog’ and being able to recognize a never before seen animal as a member of this category, we know to expect that it makes barking sounds, chases after cars and may come to lick our hand while wagging its tail. We are also able to name this new animal as ‘dog’.

Given its importance for cognition as a whole, over the past 30 years a considerable amount of research has been devoted to the development of categorization in infancy. Much of this work is based on the familiarization/novelty-preference procedure that examines infants’ abilities to rapidly form categories in laboratory-based settings. This method relies on the fact that infants tend to spend more time looking towards novel than towards familiar stimuli. In a typical categorization study, infants are familiarized to a sequence of objects from a single category and are then tested with two new objects, one from the familiarized category and one from a different category. When infants show a looking preference to the object from the new category it can be concluded that during familiarization they have formed a category that includes the new within-category object but excludes the object from the different category. For example, in a seminal study that examined the earliest perceptual categories, Quinn *et al*. [6] familiarized three- to four-month-old infants on a sequence of cat pictures and found that in the test phase they looked longer at pictures of dogs, horses and humans than at pictures of novel cats. This and related work has shown that infants as young as two months can form perceptual categories for many objects at both the basic (e.g. dogs, cats, chairs, couches) and global levels (e.g. animals, vehicles, furniture) [7–11], although global-level categories typically precede basic-level categories when compared directly [1,2,12–15].

Subsequent work has verified that the way in which these early categories are formed is contingent on the specific perceptual properties of the stimuli and even on the order in which they are presented during familiarization [16–20]. It has also become clear that previous experience interacts with the stimuli presented during testing in the laboratory. For example, Kovack-Lesh *et al*. [17] found that four-month-olds’ category formation for cats and dogs was affected by whether they had these pets at home or not.

Other work has shown that infants extract information about category prototypes from the images used during familiarization. For example, when trained on distorted triangles three- to four-month-old infants subsequently treated a prototypical (equilateral) triangle as more familiar than previously seen atypical triangles [21]. For more complex shapes only seven-month-olds showed the same effect [22]. However, the higher familiarity of a prototype after familiarization exists in parallel with item memory for atypical category members [23].

With increased age, infants’ ability to process objects becomes progressively more sophisticated, with a developing sensitivity to the correlations between object features between seven and 10 months of age. For example, Younger & Cohen [24] familiarized infants on two animal line drawings where each animal had a characteristic body shape, tail and feet. Infants were then tested on a previously seen animal, a completely novel animal and an animal that was composed of a novel combination of previously seen features (e.g. the body and feet of the first animal with the tail of the second animal). Younger and Cohen found that four-month-old infants did not look longer at this animal than at one they had seen during familiarization. By contrast, seven-month-olds looked at this new animal as long as at the animal made from completely novel features. When only two out of the three features were correlated, only 10-month-olds were sensitive to violations of feature correlations, suggesting a progressively developing sensitivity to feature correlations.

From the second half of their first year of life, infants become able to integrate different object features such as visual appearance, sounds, function and motion [25–27]. For example, although infants are sensitive to biological motion information from birth [28] and three-month-olds can discriminate familiar and novel motion paths even after a delay of one month [29], the ability to link motion information with static visual features seems to arise later in development [1,2]. In fact, in one study [30], object movement was found to interfere with infants’ learning of shape–colour compounds up to the age of 10 months. More global features indicating animacy such as eyes and fur, on the other hand, were linked to animate-like motion in seven-month-old infants [31], perhaps indicating that real-world experiences with a richer stimulus set enables learning of such associations earlier than found in the laboratory.

In a study by Madole *et al*. [25] on object function, 14-month-olds were sensitive to an object's change in function, but only by 18 months were infants able to link function with an object's static visual features. However, in other studies 11- to 12-month-olds already detected the functional relevance of object parts and categorized objects based on these parts when the function had been demonstrated to them [27], again providing evidence for a developing ability to integrate different aspects of an object and an earlier development of this ability under more ecologically valid conditions. In a related study, it was found that 10-month-olds were able to learn the relationship between an object's visual appearance and its function, but not between visual appearance and sound or between function and sound [26].

In a recent study investigating infants’ ability to learn complex crossmodal associations, Chen & Westermann [32] familiarized 10- and 12-month-old infants on two animated cartoon animals, each of which produced a characteristic unfamiliar sound. At test, both animals were shown side-by-side and one of the sounds was played. Twelve-month-olds, but not 10-month-olds, looked longer at the animal associated with the played sound, indicating that the ability to rapidly link novel objects and semantically meaningful sounds arises around 12 months of age.

Together, the described work suggests an unfolding ability of infants to learn about different aspects of objects and associate different static and dynamic features to form a complete object representation. In this way, categories in infancy are based on progressively enriched representations that integrate information from multiple sources, starting with the ability to represent individual object properties and followed by the ability to integrate these properties to form more complex representations and to detect correlations between features in one or across several sensory domains (see also [33]).

## 3. Word learning and categorization

In the second half of their first year, infants begin to learn their first words. From six months onwards, they look at the referent for some familiar nouns when spoken by their parent [34], and there is neurophysiological evidence that this understanding is referential by nine months of age [35]. In laboratory studies, infants from 13 months onwards can learn to associate novel labels with novel objects after a few familiarization trials [36]. A number of studies have asked how this emerging language affects category formation. There has been considerable debate on how early word and category learning interact. Early work by Waxman & co-workers [37–39] argued that labels act as ‘invitations to form categories’ by highlighting the common features of objects sharing a label. In several studies, infants from six to 15 months of age were familiarized on one category (e.g. dinosaurs) while either hearing a labelling phrase (‘Look, it's a toma!’), a non-labelling phrase (‘Look at this!’) or a tone sequence. At test, only infants who had heard the labelling phrase looked longer at an out-of-category item (e.g. a fish) than at a novel within-category item. However, since other studies described above had found that infants can already form perceptual categories when viewing objects in silence, it is unclear what additional role the labels played in these studies. An alternative account of these results was therefore provided by Robinson & Sloutsky [40,41], who argued that auditory information interferes with the processing of visual information but that familiar sounds such as labels disrupted visual processing less than unfamiliar sounds such as tone sequences. Nevertheless, more recent work involving a silent control condition has shown that labels can indeed override visual similarities and re-shape categories to correspond to how objects are labelled [42,43]. In one study [42], 10-month-old infants were familiarized on a continuum of eight morphed cartoon animals. Looking times at test revealed that when the stimuli were presented in silence or with a single common label, infants formed a large category comprising all eight stimuli. However, when half of the animals were labelled with one name and the other half with another, infants separated the animals into two categories according to the labels.

There has also been substantial debate over how labels affect perceived similarity in development, linked to the role that labels play in object categorization. One view holds that labels from early on act as category markers that stand as a placeholder for a concept and enable language-based inference of deeper conceptual structure [39,44,45]. A different view argues that early labels are merely another object feature in line with, for example, visual perceptual properties, and that shared labels contribute to the overall similarity between objects with the same (or indeed, similar) labels [46–50]. These two views make contrasting predictions about the interactions between visual properties and labels: according to the label-as-feature view, but not the label-as-category-marker view, shared labels should affect similarity judgements between items.

Circumstantial evidence for the label-as-feature view comes from a series of studies by Sloutsky and co-workers [47–50], but see [51,52]. For example, Sloutsky & Fisher [47] presented 4- to 5-year-old children (much older than the infants discussed so far) with picture triads containing a target item and two test options (A and B). Test option A was perceptually more similar to the target than option B. However, when target and test option B shared a label, the children judged B as more similar to the target than A.

In sum, it has become clear that labels can affect early categories by aligning category boundaries with shared labels. It also seems to be the case that shared labels affect perceived similarity of objects. However, whether labels act on the same level as visual object features or whether they are separate from visual features and act on perceptual object representations as a whole is an unresolved question.

## 4. A dual-memory model of infant categorization

As described above, many studies using the familiarization/novelty-preference procedure have found that infants can form perceptual categories at least from two months of age, and that the level of categories—basic or global—is dependent on the similarity between the specific stimuli. However, other methods that do not involve familiarization but instead assess spontaneous categorization to tap into infants’ background knowledge have often yielded quite different results [8,14]. For example, in the sequential touching paradigm, a set of toy objects from different categories is placed in front of the infant. The order in which the infant touches the toys is recorded, and above-chance touching of objects from one category is taken as evidence that the infant has categorized the objects. Using this procedure, it was found that 12- to 20-month-olds formed categories on the global level, and that basic-level distinctions within a global category were not made before 20 or even 30 months of age [53–55]. Similar results were found using the generalized imitation technique [56,57]. In this method, a certain action is modelled for the infant with a toy figure, for example, giving a dog a cup of liquid to drink. Infants are then asked to perform the same action with different toys, such as another dog, a cat or a plane. Categorization is assessed by the new toys to which the infant is extending the modelled action. It was found that 14-month-olds successfully generalized the actions to members of the same category (animals or vehicles). These results were taken as evidence that categorization is not tied to perceptual properties of objects but that it is based on conceptual knowledge and that infants generalized the modelled action to members of the same category (e.g. for a dog, another dog or a cat), rather than of the same appearance.

Given the different results from familiarization-based and non-familiarization-based studies, there has been intense debate on whether deeper concepts arise from perceptual categories through a process of enrichment [9,58,59], or whether perceptual categorization and concept learning are separate processes [8,60,61] or indeed, whether early perceptual processes are already based on conceptual object analysis [14]. We [62] presented a connectionist neural network model of infant categorization that aimed to integrate the different results from looking-time and sequential-touching/generalized-imitation-type studies. The model was based on the idea that a coherent account of this body of work could be provided by considering the unfolding interactions between two developing memory systems in the brain. In the adult literature, the view that memory formation and consolidation depend on multiple interacting neural systems is widely accepted [63,64]. One influential idea [64] is that whereas the hippocampus is responsible for rapidly acquiring new memories, cortical networks learn slowly and extract regularities from the environment. Our model was inspired by this distinction between hippocampal and cortical processing. Furthermore, it was based on the developmental literature which suggests that novelty preferences observed in infant looking-time studies are driven by the hippocampal memory system [65,66], whereas categorization behaviour displayed in imitation and object examination studies depends on a later-maturing memory network that involves inferior temporal regions [65]. The fact that different experimental methods tap into different memory representations can explain why the results in infant categorization depend on the methodology used.

**Figure 1.**
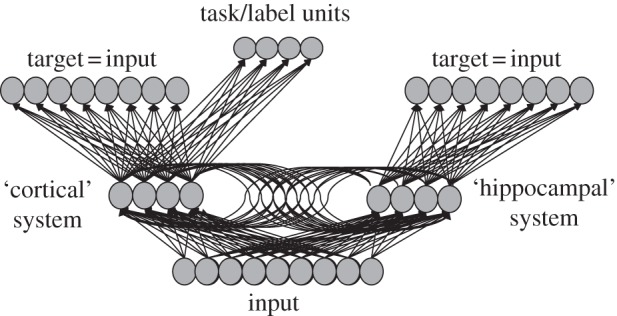
Schema of the dual-memory model.

Consequently, the model consisted of two interacting components (figure 1): a fast-learning hippocampal/striatal system and a slower learning cortical system. Each component consisted of an auto-encoder neural network: a three-layer network that receives the representation of an object as input and that learns to recreate the input on the output side. Because the hidden layer is smaller than input and output layers this creates a bottleneck, forcing the network to extract regularities from the input to solve this task. Single auto-encoder models have previously been used to model looking-time data in infant categorization [16,67–70].

In the dual-memory model (see appendix A for implementation details) the hippocampal system was used to simulate looking times in experimental situations where categories are formed online in a familiarization/novelty-preference study. The cortical component modelled the gradual development of representations that drive non-familiarization-based responses. The two systems differed only in their learning rate, that is, the rate at which weights were adapted in response to exposure to objects, with the hippocampal system adapting its connection weights at a higher rate than the cortical system. Interactions between the components were modelled by connections between the subsystems’ hidden layers. Together these architectural constraints embody a minimal set of assumptions about how the dual-memory systems can interact.

The model was trained in two ways. In *background training* objects were randomly presented to the model for random amounts of time. This training aimed to simulate infants’ experiences with objects in the real world. Modelling of category formation in familiarization studies was achieved through *familiarization training* where sequences of related stimuli were presented for fixed amounts of time. Looking times were modelled by the output of the hippocampal component: the more different the output was from the input, the longer the looking time at the stimulus [67]. Developing cortical representations were modelled by the activation patterns in the hidden layer of the cortical system.

The model was able to account for a range of results from infant category learning: in its cortical component, it learned perceptual categories that became progressively differentiated with increased exposure to exemplars, showing the global-to-basic shift that is also found in infant categories. The model could further account for the fact that background experience affects infants’ looking behaviour in an experimental setting [71]. This was because previously learned representations in the cortical system facilitated online category learning of related items in the hippocampal component through feedback connections. More recently, we used the output of the cortical system to model infant event-related potential (ERP) data in early object categorization [72].

In sum, the model covered a range of phenomena from early, prelinguistic object categorization, accounting for data from different experimental paradigms and data from behavioural and neurophysiological studies. Here, we want to extend this model to simulate the shift from prelinguistic to language-mediated categorization.

The extended model had the same architecture as the original one, but in order to model the transition from prelinguistic perceptual categorization to language-mediated categorization we extended the model with *task/label units* linked to the hidden layer of the cortical memory system (figure 1). The idea here was that these units could potentially encode a variety of functions and object properties that go beyond perceptual feature information, such as representing affordances and specific ways of interacting with an object or knowledge of an object's hidden properties. Because we aimed to model the role of first words in object categorization here these units were used to encode object labels both at the basic and global levels of categorization.

## 5. Stimuli

In order to model the development of basic- and global-level categories under varying labelling conditions, eight photographs each of objects from 26 basic-level categories were chosen. The basic-level object categories were (human) male, (human) female, dog, cat, rabbit, horse, elephant, giraffe, cow, squirrel, fish, eagle, songbird, duck, bicycle, forklift, bus, car, plane, ship, desk, table, bed, sofa, chest of drawers and chair. They fell into the four global-level categories humans, animals, vehicles and furniture that have all been previously used to test infant category formation. Exemplars varied in their within-category perceptual similarities (see appendix A).

Each of the 208 objects was represented by 18 general (geometric) and object-specific (facial) features: maximal height, minimal height, maximal width, minimal width of base, number of protrusions, maximal length/width of left, right, lower and upper protrusion, minimal width of lower protrusion, texture, eye separation, face length and face width. Feature values were scaled between 0 and 1. For each basic-level category, a prototypical object (not included in the training set) was created by averaging the feature values of all of its members.

In order to simulate an infant's general experience with the world, objects were presented in background training to the model in random order for random exposure lengths (between 1 and 1000 epochs). In simulations that explored the effect of labelling, each object had a 50% chance of being labelled, either with a global-level or a basic-level label (depending on the simulation). In these cases, the object label was presented on the task layer and connections from the hidden layer to the task layer were updated. Thereby, through training, the labels led to adjustment of the connections from the hidden to the task units. When no label was presented these connections were not updated. In the simulations reported here, each object label was represented by a single unit on the task layer. Training the model on an object proceeded as follows (for details, see appendix A): the object was presented to the input layer and activation propagated to the hidden layers. Activation then flowed between the hidden layers until they settled in a stable state. Next, hidden activations flowed to the output and, if applicable, task layers. Output values were compared with target values (the input values for the output layers and the correct label for the task layer) and all weights were updated using the backpropagation rule [73]. Weights were updated at each presentation of an object (online learning).

## 6. Results

### (a) Modelling the effects of labels on object representations

**Figure 2.**
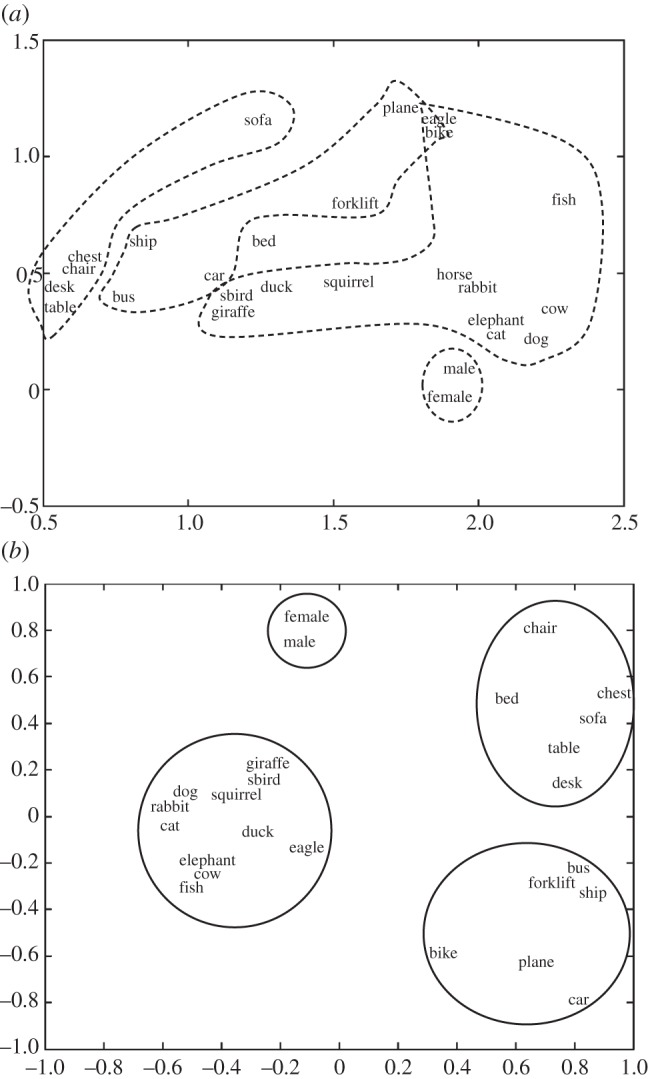
Cortical hidden layer representations of the category prototypes without labelling (*a*) and when labelled with global-level labels (*b*). The figures represent the projections of the different classes of objects onto the subspace defined by the first two principal components of the cortical hidden unit space.

The first simulation explored the effect on object representations when labelling objects with their global-level label (human, vehicle, animal, furniture). Figure 2 shows the hidden representations in the cortical system without (*a*) and with (*b*) labelling of global categories. This is a projection of the activation patterns for each category prototype of the 15 hidden units onto two dimensions using principal component analysis. When the objects were not labelled, the representations clustered on the basis of the objects’ perceptual similarities that sometimes corresponded to global-level categories (see also [62]): a cluster for mammals comprised cats, dogs, cows, elephants, horses and rabbits; a furniture cluster contained tables, desks, chairs and chests of drawers; fishes were distant from other objects but were closest to the mammals cluster. Idiosyncratic clusters such as that of plane, bicycle and eagle can be explained by the perceptual properties of these objects: in this case, each had two long, thin protrusions, wings and handlebars.

When the objects were labelled with their global-level category name (human, animal, vehicle, furniture), the internal object representations in the model were modified to separate clearly along these categories. Learning the objects’ global-level labels therefore warped the cortical representational space to reflect both perceptual similarity and the membership in different global categories. (Note that we used global-level names here for illustrative purposes to show the separation into global-level categories; infants are more likely to hear basic-level than global-level names.)

This initial result models an interaction between perceptual and language-mediated categorization: labels act on the perceptual similarity structure and warp the similarity space so that it corresponds to the labelled categories while still maintaining internal category structure. The model therefore predicts that objects sharing the same label will develop more similar representations, and thus, also be perceived as more similar. Although this result has received empirical backing [48] our model offers a different explanation to the one in the literature. As described above, a long-standing debate on this issue has yielded two conflicting accounts [47,49,51,52]: according to the label-as-feature hypothesis, a category name is merely another object feature on the same level as visual perceptual features. On this view, common labels affect the perception of overall similarity between members of a category because a shared label-feature increases the overlap between their representations. By contrast, the knowledge-based view of labels maintains that labels act as markers for objects and do not affect perceived similarity. In attaching the label on the output side of the model, the model presented here takes a middle ground between these two accounts. Here, the label is not merely a feature of the same kind as, for example, geometric extent of the object (as it is not an additional input feature). Instead the model learns a mapping between the perceptual features and the label. In this sense, the label is a concept marker and each of the members of the category evoke the label (see [74] for evidence that infants implicitly name visually fixated objects). However, differing from the knowledge-based approach, the model suggests that, to achieve this mapping, the perceptual representations of the objects are nevertheless modified and the perceptual space is warped as a consequence. Therefore, objects sharing the same label do not become more similar by virtue of merely sharing another common input feature, but because perceptual representations must be re-structured in a way that allows for the mapping between perceptual features and labels to be learned. This re-representation results in a nonlinear warping of the visual similarity space that maintains to some degree the topological relationship between the appearances of objects but reduces intra-category distance by moving objects closer to the category prototype.

### (b) Effect of word knowledge on object familiarization

In [62], we showed that, in agreement with empirical research, the model predicted faster familiarization for objects with which infants have had prior experience. Here, we investigated whether the model also predicts that knowledge of the label for a category facilitates familiarization over and above prior experience with the objects. For such background knowledge to have an effect on familiarization time, representations from the cortical component have to interact with the hippocampal representations when familiarization stimuli are presented. Depending on the developed structure of cortical representations, these could affect hippocampal processing in different ways.

**Figure 3.**
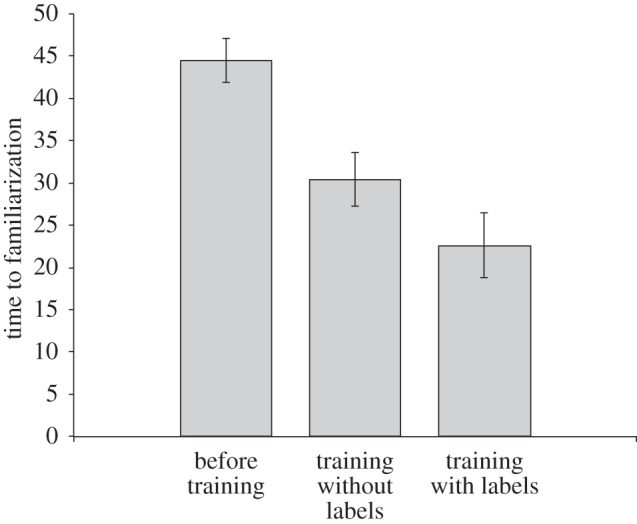
Familiarization time (measured as number of presentations until error falls below a criterion threshold) for objects (rabbits) in three models with different background knowledge (none; objects from all categories without labels; objects from all categories with basic-level labels).

We trained three models in different environments. The first model was not given experience with any background knowledge. The second model, replicating the results reported in [62], was trained on all objects from the 26 categories, but only two of the eight rabbits were used, and no object was labelled. The third model was trained like the second, but this time there was a 50% chance for each object to be labelled with its basic-level name. After training these models for 4000 object presentations, they were familiarized on the remaining six rabbits. This was done by presenting each of the rabbits to the model repeatedly until the output error of the hippocampal system fell below criterion. The results of these simulations are shown in figure 3.

Replicating the result from [62], familiarization time to the rabbit category was significantly shorter when the model had previous experience with objects (including other rabbits) than when it did not. Importantly however, when the previously experienced objects were labelled familiarization time was again significantly shorter than when they were merely presented without labels. This result predicts that infants will familiarize faster to novel exemplars of familiar objects for which they know the label than to those that are familiar but for which the label is not known. To our knowledge, this has so far not been tested with infants.

## 7. Discussion

In this paper, we first reviewed evidence of infant category development as a process that is driven by infants’ emerging abilities to integrate information about objects such as feature correlations, dynamic and animacy cues, sounds and labels. We then extended an artificial neural network model that had previously been used to simulate prelingusitic category learning to model the effect of labels on categorization. The model suggested that labels warp the visual representational space so that objects from the same category are represented as more similar to each other. The model further predicts that through the interaction between cortical and hippocampal memory systems, knowing the label for a familiar object will speed up familiarization to other exemplars of this object category in looking-time tasks compared with a familiar object for which the label is not known.

The model makes a number of contributions. First, it provides a new perspective on the debate surrounding whether labels act as object features or as category placeholders. Although in the model shared labels increase the similarity between objects, which has been a prediction of the label-as-feature view, in the model the label is not a feature but acts more like a category marker. The model, which implements the view of category learning as a continuous process based on the progressive enrichment of object representations ([9], see also [75]), therefore suggests how labels as category markers, which have in experimental studies been found to affect category formation, can interact with and reorganize prelinguistic representations.

Second, the model offers an integrated view of prelinguistic categorization, where it has accounted for looking time data from infant studies and the global-to-basic shift, and the effect of labels on categorization, where it simulates results showing that labels can shape category structure and that a common label makes objects appear more similar.

Several related models have addressed early categorization and word learning. For example, a recent connectionist model of word learning [76] consisted of two maps, one visual and one for labels, and learning label-object mappings was achieved by linking units between the maps. Although the model was able to account for phenomena from word learning such as taxonomic responding, a vocabulary spurt and overextensions, it had to make a number of assumptions that are in contrast to empirical evidence. The model required that both word and visual maps were fully established before mappings between them could be formed, and the associations did not affect the representations on each map. It is, however, unlikely that infants have achieved a considerable vocabulary prior to linking any words to objects. Furthermore, as described above, there is considerable evidence that mappings between objects and labels affect their representations (see also [77,78]). This model therefore accounted neither for prelinguistic categorization nor for the effect of labels on categories.

A related model [79] took the opposite label-as-feature approach so that visual features and labels together fed into a common category map. This model was successful in accounting for data from the effect of labels on category formation, but the dual-memory model goes beyond that model in that it can additionally account for looking-time data in prelinguistic categorization as well as for the global-to-basic shift in non-familiarization-based categorization studies.

Our model is perhaps most closely related to that of Rogers & McClelland [80]. This model maps between objects and their perceptual and non-obvious properties. When being trained on objects from multiple global- and basic-level categories, the model showed a global-to-basic shift in category differentiation as well as aspects of word learning such as label overextensions. However, their model focused on learning in what in our model is the cortical component and it did not attempt to provide an integrated account of real-world learning and online learning in looking-time tasks. Instead it focused on those categorization studies that do not involve familiarization. The model therefore could not account for effects of labels on category structures found in the described laboratory-based studies. In contrast to our model, however, this model accounted for several aspects of adult categorization and of semantic dementia.

Clearly, these models are complementary in the phenomena they address, and an integrated model should account for different aspects of acquisition together with normal and impaired adult performance (for a related point, see [81]). Furthermore, following a neuroconstructivist approach that views typical and atypical development within the same framework [82–84], a comprehensive model should also aim to account for categorization in children with developmental disorders. Studies on different disorders suggest that the early processes described in this paper might be disrupted, leading to cascading deficits that manifest later in life. For example, one study with 4- to 6-year-old children with Williams syndrome (WS) found that these children were able to categorize objects based on similarity, but they were unable to categorize on the basis of shared labels, despite having developed large vocabularies [85]. This deficit to use verbal labels as category markers might itself be a downstream consequence of WS children's difficulty in planning visual saccades and thus, triadic interactions [86]. In WS, therefore, at least the saliency of the inputs to our model would be altered to reduce the ability to link object representations with labels.

Evidence for atypical categorization and word learning has also been found in studies with children with autism spectrum disorder (ASD). Children and adults with ASD respond more slowly to atypical category members in categorization tasks than healthy controls do [87]. Furthermore, individuals with ASD may have difficulty in forming prototypes when shown varying exemplars from a category [88,89], an ability that is already evident in typically developing three-month-olds. These results suggest a perceptual processing deficit that goes beyond attending to stimuli differently. In our model, categorization and prototype formation are automatic processes that arise from the type of learning in connectionist models. A model of categorization in autism would therefore need to modify the central aspects of information processing in the model. Several accounts of the observed perceptual deficits in ASD that rely on abnormal neural processing have been put forward that could beneficially be explored in extensions of the current model [90–92].

Finally, children with word-finding difficulties (WFD) present an interesting case to the model. These children have dissociations between word comprehension and production, with more difficulty in production compared with age-matched peers [93]. There is a debate over whether the core deficit in WFD is a semantic one (such as weak links between semantically related concepts) or a phonological one (such as impoverished phonological representations) [93]. In our model, there are multiple loci where impairment could be simulated: sematic deficits can arise from imprecise featural object descriptions as input to the model or from impaired semantic processing, for example, through noisy connections in the network. The most direct simulation of WFD without semantic deficits would consist in damaging connections between the cortical hidden layer and the task/label units. The challenge is to build a developmental model that can provide insights into whether damage to the different parts of the model leads to performance deficits that are comparable to those in WFD.

Our model as presented here is only the first step of an integrated view of category and concept development. We have focused on early categorization from two months to around 2 years of age. However, in the transition from categorization to concept formation, labels can take a role above merely re-shaping perceptual categories. Many studies have shown that labels can serve as the basis for inferences about an object's non-obvious properties where they can override perceptual similarities ([48], e.g. [94–96]). Furthermore, recent priming studies have shown that at least from 18 months onwards there are close links between category representations and the phonological representations of their labels [74], as well as semantic representations and labels [97,98]. We do not believe that our model is incompatible with this research and we plan to extend it in this direction to provide a comprehensive account from early categorization to concept formation.

## Funding statement

G.W. was supported by ESRC grant RES-000-22-4470 and by a Lichtenberg Fellowship from the University of Göttingen, Germany. D.M. was supported in part by a Royal Society-Wolfson research merit award.

## Appendix A

### A.1. Model details

The model had 18 input units, 15 hidden units each and 18 output units each in the hippocampal and the cortical systems. For the simulation in which global-level labels were used it had four task/label units, and for the simulation with basic-level labels it had 26, one for each category. Layers were fully interconnected with unidirectional feed-forward connections. The two hidden layers were fully interconnected with lateral connections (i.e. the hippocampal hidden units projected into the cortical hidden layer and vice versa). Weights were initialized in the range ±0.5. Training proceeded as follows: an input was presented to the model and activation flowed to both hidden layers. Then, activation flowed between the hidden layers (with inputs clamped) until a stable activation state was reached, i.e. until the activation change for any hidden unit was less than a threshold (0.01), or for a maximum of 50 iterations. In the next step, activation flowed from the hidden layers to the three output layers. Errors were computed on all units according to the backpropagation rule. Weights were updated with simple backpropagation with a ‘Fahlman-offset’ of 0.1 [99] and a momentum of 0.9. Learning rates were as follows: in the hippocampal subsystem, 0.25; in the cortical subsystem, 0.01; lateral connections from cortical to hippocampal hidden layer, 0.1; lateral connections from hippocampal to cortical hidden layer, 0.01; task unit connections, 0.01.

### A.2. Category structure

Category compactness was computed by calculating the mean Euclidean distance between all members of a category (each expressed as an 18-dimensional feature vector). These were: female: 0.4556; male: 0.7204, dog: 0.8501; cat: 0.9072; rabbit: 0.8001; horse: 0.8085; elephant: 0.7532; giraffe: 0.5951; cow: 0.8259; squirrel: 0.8332; fish: 0.8796; eagle: 1.3012; songbird: 0.6807; duck: 0.6756; desk: 0.4585; table: 0.2619; bed: 0.6352; sofa: 0.3459; chest of drawers: 0.6014; chair: 0.2262; bike: 0.5659; forklift truck: 0.5128; bus: 0.4160; car: 0.2953; plane: 0.9548; ship: 0.4402. In the interests of brevity, further details of the stimulus materials have been omitted here, but are available by request to the authors.
